# Draft genome sequence of *Pestalotiopsis disseminata* HY98, isolated from diseased *Euonymus japonicus* leaves in China

**DOI:** 10.1128/mra.00319-26

**Published:** 2026-06-22

**Authors:** Tan Wang, Zhanying Zhu, Zimeng Kou, Qiuhong Niu, Sengen Zhu

**Affiliations:** 1 School of Life Science, Nanyang Normal University 71072, Nanyang, China; 2 Zhejiang Honggaitou Agricultural Science and Technology Co., Ltd, Quzhou, China; University of Strathclyde, Glasgow , United Kingdom

**Keywords:** genome sequence, *Pestalotiopsis disseminata*

## Abstract

We report the 46.7-Mb draft genome sequence resource for *Pestalotiopsis disseminata* strain HY98, isolated from symptomatic leaves of *Euonymus japonicus* in China. The assembly comprises seven contigs with 64× coverage and provides a useful foundation for comparative analyses of host association, genome evolution, and plant interactions.

## ANNOUNCEMENT

*Pestalotiopsis disseminata* is a plant-associated coelomycetous fungus that has been reported from several woody hosts, including *Eucalyptus* spp. and stone pine (*Pinus pinea*) (1–4); however, genomic resources for the genus remain limited (5, 6). To support comparative genomic analyses and future investigations into pathogenicity and host interactions, we generated a genome resource for strain HY98, which was originally isolated by our group from symptomatic leaves of *Euonymus japonicus* collected in Henan Province, China (32°30′58″ N, 112°19′44″ E) (7). The isolate was subsequently deposited in the publicly accessible collection of the China General Microbiological Culture Collection Center (CGMCC) under accession number CGMCC 3.29727, with no restrictions on access. Diseased leaf tissues were surface disinfected with 1% NaClO for 1 min, rinsed three times in sterile water, excised into small segments, and plated onto potato dextrose agar (PDA) (Sangon Biotech, Shanghai, China) amended with streptomycin. Following incubation at 25°C, representative colonies were purified and maintained on PDA slants at 4°C and subcultured every 3 months. Strain HY98 was previously identified as *P. disseminata* (8, 9).

Prior to genomic DNA extraction, the preserved culture was revived on fresh PDA plates at 25°C for 5 days. Genomic DNA was extracted using the Fungal Genomic DNA Extraction Kit (Tiangen Biotech, Beijing, China), and DNA quality was assessed by 1% agarose gel electrophoresis. High-molecular-weight DNA was sheared to approximately 15 kb with g-TUBE devices (Covaris, USA). A long-insert SMRTbell library was prepared using the SMRTbell Express Template Prep Kit 2.0 (Pacific Biosciences, Menlo Park, CA, USA) according to the manufacturer’s instructions (9), and target-size fragments of approximately 15 kb were selected and purified using the SageELF system. Sequencing was performed on the PacBio Sequel IIe platform (Pacific Biosciences) using Sequel II Binding Kit 2.0 and SMRT Cell 8M chemistry, and HiFi reads were generated after quality filtering using the built-in SMRT Link (version 13.1) workflow for downstream assembly and analyses. In total, 162,306 HiFi reads totaling 3,010,068,302 bp were obtained, with a read N50 of 18,932 bp. HiFi reads were assembled *de novo* using Hifiasm (version 0.19.9) (10). Redundant haplotypic sequences were removed with Purge_dups (version 1.2.6) (11) after aligning HiFi reads to the assembly using Minimap2 (version 2.28) (12). Unless otherwise noted, default parameters were used for all software.

The final assembly consisted of seven contigs, with an N50 of 6,426 kb, a total assembly size of 46,675,635 bp, and a GC content of 51.97%. PacBio long-read data provided approximately 64× coverage of the 46.7-Mb genome. Assembly completeness was assessed using BUSCO (version 3.0.2) (13) with the sordariomyceta_odb9 reference data set, which identified 758 complete BUSCO genes (99.2%), indicating a highly complete genome assembly. Protein-coding genes were predicted using AUGUSTUS (version 3.5.0) (14), SNAP (version 2006-07-28) (15), GlimmerHMM (version 3.0.4) (16), and GeneMark-ET (version 4.71) (17), and integrated with EVidenceModeler (version 1.1.1) (18); default parameters were used for all software. A total of 13,282 protein-coding genes were predicted. This assembly provides a high-quality genomic resource for comparative analyses within the genus *Pestalotiopsis* and will support future investigations into host association, genome evolution, and candidate genes involved in plant interactions.

## Data Availability

This Whole Genome Shotgun project has been deposited in DDBJ/ENA/GenBank under accession number JBVVWU000000000. The version described in this paper is version JBVVWU010000000. The associated BioProject, BioSample, and Sequence Read Archive accession numbers are PRJNA1356826, SAMN53068702, and SRR36288240, respectively. Genome annotation files generated using EVidenceModeler, including GFF3 annotation files and predicted protein sequences, have been deposited in Zenodo and are publicly available at https://doi.org/10.5281/zenodo.20199242.
